# Distinct patterns of whole blood transcriptional responses are induced in mice following immunisation with adenoviral and poxviral vector vaccines encoding the same antigen

**DOI:** 10.1186/s12864-021-08061-8

**Published:** 2021-10-30

**Authors:** Dylan Sheerin, Christina Dold, Daniel O’Connor, Andrew J. Pollard, Christine S. Rollier

**Affiliations:** 1 Oxford Vaccine Group, Department of Paediatrics, University of Oxford, and the NIHR Oxford Biomedical Research Centre, Centre for Clinical Vaccinology and Tropical Medicine, Churchill Hospital, Oxford, UK; 2 Infectious Diseases and Immune Defence Division, The Walter & Eliza Hall Institute of Medical Research (WEHI), Melbourne, Victoria 3052 Australia

## Abstract

**Background:**

Viral vectors, including adenovirus (Ad) and modified vaccinia Ankara (MVA), have gained increasing attention as vaccine platforms in recent years due to their capacity to express antigens from a wide array of pathogens, their rapid induction of humoral and cellular protective immune responses, and their relatively low production costs. In particular, the chimpanzee Ad vector, ChAdOx1, has taken centre stage as a leading COVID-19 vaccine candidate. However, despite mounting data, both clinical and pre-clinical, demonstrating effective induction of adaptive immune responses, the innate immune signals that precede the protective responses that make these vectors attractive vaccine platforms remain poorly understood.

**Results:**

In this study, a mouse immunisation model was used to evaluate whole blood gene expression changes 24 h after either a single dose or heterologous prime-boost regimen of an Ad and/or MVA vaccine. We demonstrate through comparative analysis of Ad vectors encoding different antigens that a transgene product-specific gene signature can be discerned from the vector-induced transcriptional response. Expression of genes involved in TLR2 stimulation and γδ T cell and natural killer cell activation were induced after a single dose of Ad, while MVA led to greater expression of type I interferon genes. The order of prime-boost combinations was found to influence the magnitude of the gene expression changes, with MVA/Ad eliciting greater transcriptional perturbation than Ad/MVA. Contrasting the two regimens revealed significant enrichment of epigenetic regulation pathways and augmented expression of MHC class I and II molecules associated with MVA/Ad.

**Conclusion:**

These data demonstrate that the order in which vaccines from heterologous prime-boost regimens are administered leads to distinct transcriptional responses and may shape the immune response induced by such combinations. The characterisation of early vaccine-induce responses strengthens our understanding of viral vector vaccine mechanisms of action ahead of their characterisation in human clinical trials and are a valuable resource to inform the pre-clinical design of appropriate vaccine constructs for emerging infectious diseases.

**Supplementary Information:**

The online version contains supplementary material available at 10.1186/s12864-021-08061-8.

## Background

Viral vectors have gained prominence as vaccine platforms, with several species of viruses being assessed in animal models and in clinical trials. The majority of these candidates are based on various serotypes of adenovirus (Ad). Ad vectors represent a particularly attractive platform for vaccine development owing to the fact that they stimulate strong CD8^+^ T cell responses, of particular importance when dealing with pathogens that exhibit intracellular replication cycle stages [1]. This characteristic has made them a prominent choice as candidate vaccines for the prevention of malaria [2], human immunodeficiency virus (HIV) [3], and hepatitis C virus (HCV) [4], and more recently as vaccines authorized for use in humans to protect against Ebola virus [5, 6] and SARS-CoV-2 [7]. Ad vectors are also effective at eliciting humoral immunity, by stimulating durable antigen-specific germinal centre responses on par with the best available protein-in-adjuvant formulations [8]. Their ability to induce high titres of protective antibody in humans has been showcased by several Ad-based Ebola virus vaccine candidates that demonstrated immunogenicity in outbreak and non-outbreak settings [9, 10]. The development of replication-defective chimpanzee-derived Ad vectors, that the vast majority of humans have never been exposed to making them significantly less susceptible to antibody neutralisation [11], has resulted most recently in one of the leading vaccine candidates against coronavirus disease 2019 (COVID-19). The Ad-based vaccine, ChAdOx1-nCoV-19, has been shown to induce neutralising antibody titres and antigen-specific T cell responses in clinical trials in humans [12–14]. Ad vaccines have been successfully utilised in conjunction with the modified vaccinia Ankara (MVA) poxvirus vector as part of heterologous prime-boost regimens, that increase the T-cell response while circumventing potential issues with vector-specific immune responses directed at the prime vaccine. Enhanced CD8^+^ T cell responses to intracellular pathogens such as *Plasmodium falciparum*, HCV and HIV [15–19], have been demonstrated in humans. While numerous clinical studies have demonstrated the immunogenicity of Ad and MVA vaccines, administered separately and in combination, surprisingly little is known about the innate immune responses induced by either vector.

Several studies have employed RNA-sequencing (RNA-seq) to explore the early transcriptional changes following immunisation with experimental and established vaccines, such as the RTS,S malaria vaccine [20], influenza vaccines [21–23], and conjugate vaccines [24]. This approach has also been extended into mice for the exploratory analysis of different vaccine platforms and components through whole blood transcriptomics, for example for the acellular pertussis vaccine and pertussis outer membrane vesicles (OMVs) [25, 26], and a variety of novel vaccine adjuvants [27]. We previously applied comparative transcriptomics to identify molecular determinants of vaccine-induced reactogenicity within the first 24 h post-immunisation in mice and infants [28, 29]. There is a paucity of data related to the transcriptional responses induced by viral vector vaccines. The transcriptional response to a candidate recombinant vesicular stomatitis virus (rVSV)-based Ebola virus vaccine was analysed using RNA-seq on samples taken from human participants during a phase I trial, to establish an early innate immune signature as a potential correlate of protection [30]. Transcriptomic analyses have also been applied to simian Ad serotypes, with one study involving the immunisation of humans with ChAd3- or AdC63-vectored HCV or HIV-1 immunogens, respectively [31]. Participants in this study were also boosted with an MVA vaccine and transcriptional changes were assessed using microarray technology. RNA-seq was utilised in the present study to determine the early gene expression changes (24 h post-immunisation) in whole blood after immunising mice with human Ad serotype 5 (HuAd5)- and MVA-based pre-clinical vaccine constructs with the aim of characterising the immunological pathways that underlie their respective mechanisms of action. We compared two different HuAd5 constructs, one encoding a bacterial antigen (the meningococcal factor H binding protein [fHbp] and one encoding a viral antigen (the respiratory syncytial virus [RSV] pre-fusion F protein [preF], with an HuAd5 construct containing no transgene sequence and with each other to determine whether specific gene expression signatures could be detected and distinguished between vaccine-encoded antigens. Transcriptional responses induced by a single dose of an fHbp-expressing MVA construct were also defined. Finally, to better understand how the immune system responds to a heterologous prime-boost of two distinct viral vector vaccine platforms, and how the order in which they are administered influences that response at a transcriptional level, we performed a pathway enrichment analyses on samples taken 24 h after the final dose of each of two heterologous prime-boost regimens: Ad prime, MVA boost; MVA prime, Ad boost. The data presented characterise both vaccine-encoded antigen- and viral vector-specific host responses using high-throughput sequencing to inform the selection of appropriate vaccine platforms and combinations to elicit the desired immune responses for a given disease.

## Results

### Adenovirus vector-encoded antigen-specific transcriptional responses are detectable in mouse whole blood 24 h post- immunisation

**Fig. 1:**
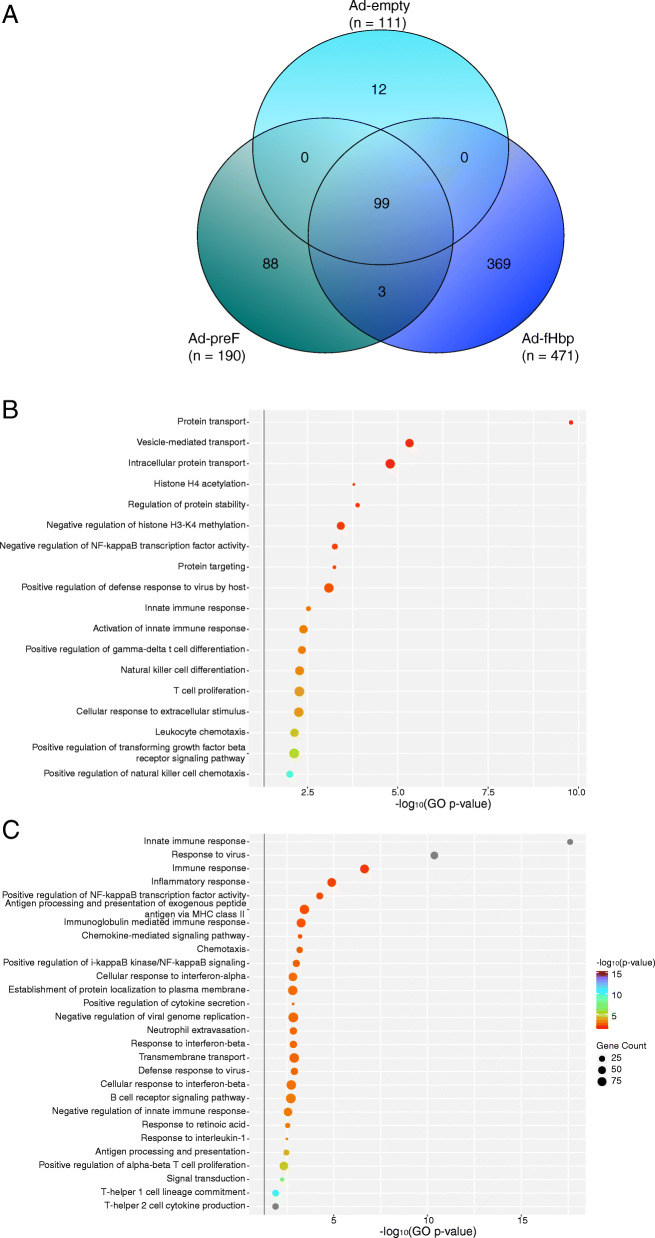
Significantly differentially enriched gene ontologies in mice associated with the expression of transgene-encoded antigens from an adenovirus vector. **a** Overlap between significantly differentially expressed genes (DEGs) in mouse whole blood 24 h after immunisation with human adenovirus serotype 5 (HuAd5) encoding a bacterial (factor H binding protein, Ad-fHbp), viral (pre-fusion F protein, Ad-preF), or no antigen (Ad-empty). Significantly DEGs were defined as those with an FDR < 0.01. Genes unique to each vaccine group represent putative transgene-encoded antigen-specific genes, while genes in the intersection between all three groups represent HuAd5 vector-specific genes. Gene ontology (GO) over-representation analysis was performed on significantly DEGs associated with (**b**) the Ad-fHbp vaccine and (**c**) the Ad-preF vaccine, defined relative to the Ad-empty group. The colour and position of circles along the x-axis corresponds to the negative log_10_ *p*-value associated with a given GO (y-axis), while the size of the circle corresponds to the number of genes defined for that GO. The horizontal grey line indicates the threshold for significant (*p*-value < 0.05)

Whole blood gene expression changes elicited by the viral vector transgene product were determined by comparing RNA-seq data obtained from the peripheral blood of mice immunised with HuAd5 vector vaccines encoding the meningococcal fHbp antigen (Ad-fHbp), the pre-fusion form of the F protein from RSV (Ad-preF), or an HuAd5 vector lacking any transgene sequence and therefore expressing no exogeneous antigen (Ad-empty). Firstly, significantly differentially expressed genes (DEGs, false discovery rate-adjusted *p*-value < 0.01) were defined for each of the three Ad-immunised groups individually by contrasting each against the naïve control group. The overlap between significantly DEGs for each group was determined, revealing an overlap of 99 significant genes between all three Ad groups and therefore likely representing the HuAd5-vector specific response (Fig. 1a). There were 369 and 88 uniquely differentially expressed, and therefore putatively transgene product-specific, genes associated with the Ad-fHbp and Ad-preF groups, respectively (Fig. 1a). To discern the antigen-specific gene expression changes associated with the preF and fHbp transgene-encoded antigens, contrasts were defined between the Ad-fHbp or Ad-preF groups and the Ad-empty group. Linear models were fitted for these contrasts and then assessed for differential expression using empirical Bayes testing to identify significant DEGs. Gene ontology (GO) over-representation analysis (ORA) was then performed on these distinct gene lists to determine significantly differentially enriched ontologies (pathway *p*-value < 0.05) for each vaccine (Fig. 1b). Three protein transport pathways – protein binding (GO:0005515), vesicle-mediated transport (GO:0016192) and intracellular protein transport (GO:0006886) – were identified among the top differentially enriched GOs induced following immunisation with HuAd5 expressing fHbp, indicating the induction of transgene product-specific cellular processing pathways. Additionally, several immune cell population ontologies were differentially enriched, including γδ T cell differentiation and natural killer cell differentiation and chemotaxis pathways. This suggests that transgene expression also leads to immune cell engagement that may be specific to the fHbp antigen. Several epigenetic modification ontologies, including histone H4 acetylation and negative regulation of methylation of lysine (K)4 on histone H4. The Ad-preF GO ORA revealed significant upregulation of viral innate immune response, antigen processing and presentation, and immune cell ontologies including B cell activation, neutrophil extravasation, and enrichment of T cells of the T_H_1 and T_H_2 lineages, relative to the Ad-empty group (Fig. 1c). This analysis implies a transgene-encoded antigen-specific response associated with the preF that differs from that of fHbp in the nature of the innate immune response, viral compared with bacterial, and a pattern of immune cell gene expression expression that indicates the involvement of neutrophils and αβ T cells rather than natural killer cells and γδ T cells.

**Fig. 2:**
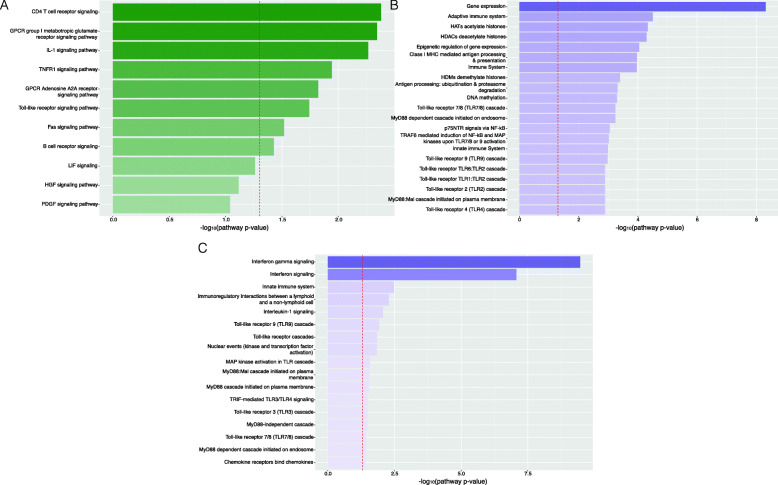
Significantly differentially enriched immunological and cell signalling pathways in mice associated with transgene-encoded antigen expression from an adenovirus vector. Pathway over-representation analysis was performed on differentially expressed genes induced 24 h post-immunisation with a human adenovirus serotype 5 (HuAd5) encoding a bacterial (factor H binding protein, Ad-fHbp) or viral (pre-fusion F protein, Ad-preF), relative to HuAd5 encoding no antigen (Ad-empty). The enrichment of genes induced by Ad-fHbp pertaining to pathways categorised in the publicly available (**a**) integrating network objects with hierarchies (INOH) cell signalling pathway and (**b**) Reactome immunological pathway databases was assessed. **c** Immunological pathway enrichment was also assessed for Ad-preF. The x-axes correspond to the negative log_10_ pathway *p*-value associated with each pathway (y-axes). The dashed red lines correspond to a negative log_10_ pathway p-value cut-off of 0.05

A separate pathway ORA was also performed on significantly DEGs identified for the Ad-fHbp and Ad-preF vaccines to elucidate the immunological and cell signalling pathways specifically induced by fHbp or preF when expressed from Ad. The former revealed the ‘gene expression’ pathway to be substantially enriched in the Ad-fHbp group relative to the Ad empty group, likely due to the large quantities of transgene expressed from Ad vectors, as well as several pathways related to epigenetic regulation, from histone acetylation and deacetylation to DNA methylation and demethylation (Fig. 2a). Further to this, the immunological pathway ORA also revealed the specific TLR pathways induced by fHbp produced from the Ad vector. These include stimulation of TLR2 and its complexes, TLR1:TLR2 and TLR6:TLR2, characteristic of an innate response to lipoprotein, as well as TLR4 engagement and the downstream MyD88:Mal cascade. Cell signalling pathway ORA revealed significant enrichment of CD4+ T cell signalling as well as IL-1 signalling, TNF receptor engagement, and TLR and BCR signalling following Ad-fHbp immunisation (Fig. 2b). The Ad-preF pathway ORA revealed no significant differentially enriched cell signalling pathways relative to the Ad-empty group. However, immunological pathway ORA further delineated aspects of the putative antigen-specific immune response, including enriched interferon signalling and engagement of TLRs 3, 7, 8, and 9 (Fig. 2c). The TLR cascades initiated by the expression of the preF antigen are in contrast with those initiated by fHbp expression – TLR2, TLR1:2, and TLR6:2. These pathway enrichment analyses demonstrate the high specificity and resolution of RNA-seq for defining transgene product-specific perturbations to the whole blood transcriptome.

### The antigen-specific transcriptional response associated with two distinct adenovirus-encoded antigens can be distinguished through comparative assessment of whole blood gene signatures

**Fig. 3:**
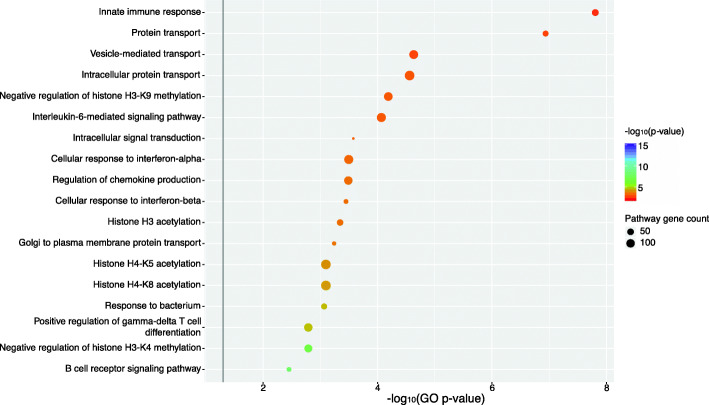
Transgene-encoded antigen-specific gene ontologies differ significantly between a bacterial and viral transgene product expressed from the same adenovirus vector. Gene expression profiles defined in mice 24 h after immunisation with a human adenovirus serotype 5 vector encoding either a bacterial (factor H binding protein, Ad-fHbp) or viral (pre-fusion F protein, Ad-preF) antigen were contrasted against each other to determine significantly differentially expressed genes (DEGs). Gene ontology (GO) over-representation analysis was performed on these DEGs to determine significantly differentially enriched GOs. The colour and position of circles along the x-axis corresponds to the negative log_10_ *p*-value associated with a given gene ontology (y-axis), while the size of the circle corresponds to the number of genes defined for that ontology. The horizontal grey line indicates the threshold for significant (pathway *p*-value < 0.05)

To determine whether the gene expression changes and differentially enriched pathways identified for each of the transgene-encoding vectors were related specifically to the expression of the transgene product, the Ad-fHbp and Ad-preF groups were directly contrasted in the same manner as the Ad-fHbp versus Ad-empty comparison. Given that the Ad-fHbp vaccine induced a greater magnitude of gene expression changes than the Ad-preF vaccine, a greater proportion of ontologies and pathways were significantly enriched in the Ad-fHbp group relative to the Ad-preF group. A GO ORA of genes found to be significantly differentially expressed between these two vaccine groups further emphasised the enrichment of transgene product-specific immune responses defined for Ad-fHbp (Fig. 3). Ad-fHbp-induced gene expression changes were also enriched for several GO terms denoting epigenetic modification including the negative regulation of K4 and K9 methylation on histone H3 and acetylation of histone H3 and K5 and K8 on histone H4. Ad-fHbp also engages gene expression programmes implicated in the differentiation of γδ T cells while Ad-preF does not.

### Adenovirus and MVA viral vectors exhibit differential engagement of immunological pathways despite encoding the same transgene

Having characterised the response to Ad-fHbp, a similar analysis was performed for an MVA vector encoding the same antigen to determine vector-specific gene expression changes prior to exploring heterologous prime-boost combinations of these vaccines. Gene expression changes in whole blood were defined 24 h after intramuscular immunisation with a single dose of MVA-fHbp. A greater number of significantly DEGs were obtained following Ad-fHbp immunisation than MVA-fHbp immunisation (471 compared with 168) despite the larger size and repertoire of antigens associated with MVA vectors. The common significant DEGs in the intersection between these two vaccine groups are predominantly related to the shared antiviral responses and include various IFN-induced and IFN regulatory factor genes (*Isg15*, *Irf7*, *Ifit1*), 2′-5′-oligoadenylate synthetase genes, (*Oas3*, *Oasl2*, *Oasl1*), guanylate-binding protein genes (*Gbp7*, *Gbp2*, *Gbp6*), and the gene encoding the RNA-sensing TLR7 (*Tlr7*). Furthermore, of the 168 significantly DEGs associated with MVA-fHbp, 82 were found to overlap with all three Ad groups (fHbp, pre-F and empty) and may therefore be common antiviral response genes, while 57 (approximately one third) were commonly expressed between Ad-fHbp and MVA-fHbp (Fig. S1). These genes may represent the core fHbp-specific genes that are induced irrespective of the vector from which the antigen is expressed and include *Il27*, *Il15*, *Fcgr4*, *Fcgr1*, *Ccrl2*, *Ly6a, Tlr7*. It is possible that the remaining significantly DEGs exclusive to Ad-fHbp may be a factor of the transgene expression kinetics and the immunodominance of the encoded antigen relative to the vector antigens.

**Fig. 4:**
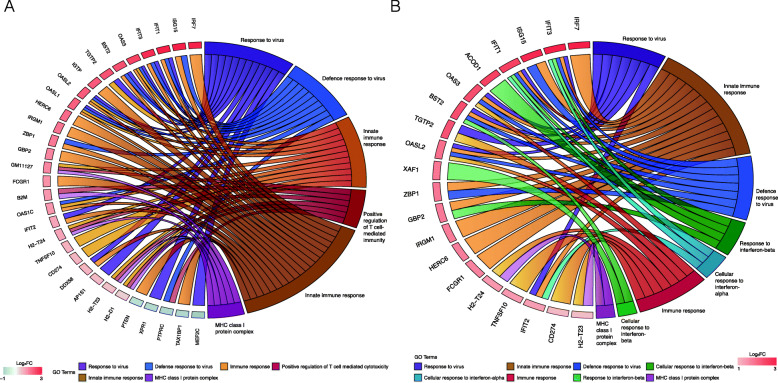
Chord plots of top significantly enriched gene ontologies associated with adenovirus and modified vaccinia Ankara immunisation in mice. Significantly differentially expressed genes (DEGs) (FDR < 0.05) associated with a single dose of human adenovirus serotype 5 or modified vaccinia Ankara encoding a factor H binding protein transgene (Ad-fHbp and MVA-fHbp, respectively), 24 h after a single dose were analysed for enrichment of gene ontology (GO) categories. GO categories containing greater than three of the top significantly DEGs were included in the chord plots and their names are displayed along the right hemisphere of each plot. The names of the proteins encoded by the corresponding DEGs were ranked according to the gene’s log_2_ fold-change along the left hemisphere, from highest to lowest. **a** Ad-fHbp. **b** MVA-fHbp

A side-by-side comparison of gene ontologies between the Ad-fHbp and MVA-fHbp vaccine groups highlighted a largely similar pattern of immunological pathway enrichment, with both vaccines eliciting a classical antiviral response characterised by induction of IFN regulatory factor (IRF), IFN-induced transcript (IFIT), and oligoadenylate synthase (OAS) family genes (Fig. 4a, b). The MHC class I protein complex, commonly enriched in both groups, is the primary mediator of antigen presentation from these vectors, but Ad-fHbp immunisation promotes greater enrichment of genes involved in T cell-mediated responses (Fig. 4a). On the other hand, MVA-fHbp immunisation leads to greater expression of genes that promote the activity of type I IFNs, IFN-α and IFN-β (Fig. 4b).

### Differential upstream gene regulation following heterologous boost of viral vector vaccine combinations influences immunological pathway engagement

**Fig. 5:**
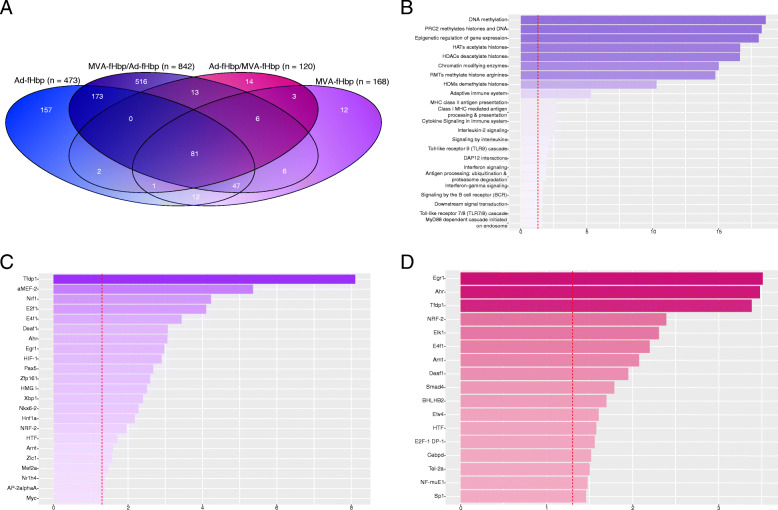
A side-by-side assessment of significantly over-represented transcription factor binding site pathways associated with the immunisation of mice with one of two heterologous viral vector vaccine prime-boost combinations, 24 h after the boost dose. **a** Overlap between significantly differentially expressed genes in mouse whole blood 24 h after immunisation with one of a series of candidate viral vector vaccines, administered as a single dose or heterologous prime-boost combination. Significantly differentially expressed genes (DEGs) were defined as those with an FDR < 0.01. The intersection between the two ovals indicates common DEGs between the two conditions. The number of significantly DEGs in each group is denoted by “n”. **b** Gene expression changes induced in mice 24 h after the boost dose of either a human adenovirus serotype 5 vaccine encoding a factor H binding protein (fHbp) transgene (Ad-fHbp) prime, modified vaccinia Ankara (MVA)-fHbp boost, or an MVA-fHbp prime, Ad-fHbp boost heterologous vaccine combination were contrasted to determine significantly DEGs associated with each vaccine combination. Pathway over-representation analysis was performed on these DEGs to determine the enrichment of transcription factor binding site pathway categories from the publicly available CisRED database. **c** MVA-fHbp prime, Ad-fHbp boost. **d** Ad-fHbp prime, MVA-fHbp boost. The x-axes correspond to the negative log_10_ pathway *p*-value associated with each pathway (y-axes). The dashed red lines correspond to a negative log_10_ pathway *p*-value of 0.05

Significantly DEGs (FDR < 0.01) were determined for the two heterologous prime-boost vaccine combinations – MVA-fHbp prime, Ad-fHbp boost (MVA-fHbp/Ad-fHbp) and Ad-fHbp prime, MVA-fHbp boost (Ad-fHbp/MVA-fHbp) – 24 h after the booster dose of vaccine, administered 8 weeks after the prime. The MVA-fHbp/Ad-fHbp combination induced the greatest perturbation of the mouse whole blood transcriptome, with 842 significantly DEGs relative to the naïve control group, while the Ad-fHbp/MVA-fHbp induced even fewer significantly DEGs than a single dose of MVA-fHbp, with just 120 genes passing the significance threshold (Fig. 5a). MVA-fHbp appears to induce relatively mild transcriptional perturbation, even when administered as a heterologous boost. Ad-fHbp, on the other hand, induces an even greater transcriptional response when administered as a heterologous boost.

Comparative assessment of the heterologous prime-boost vaccine combinations revealed substantial enrichment of immunological pathways related to epigenetic modification associated with the MVA-fHbp/Ad-fHbp combination; these pathways were predominant among the top significantly enriched pathways based on contrasts drawn between these two groups (Fig. 5b). Additionally, the MVA-fHbp/Ad-fHbp group displayed greater enrichment of antigen processing and presentation pathways related to both MHC class I and II molecules, indicating differential propensities to express and present exogenous antigen at this timepoint.

To assess the impact that these alterations may have on the accessibility of transcription factor binding sites (TFBS), an ORA of experimentally-validated TFBS from the publicly available CisRED regulatory elements database was performed based on expression of corresponding target genes identified for each prime-boost combination group (Fig. 5C, D) [32]. Among the biologically relevant TFBS enriched specifically in the Ad-fHbp boost group were the following:

- PAX5, a transcription factor involved in B cell differentiation [33] and known to undergo frequent methylation in the context of cancer [34].
- XBP1, a transcription factor that plays a key role in the secretory apparatus of plasma cells and DCs when chromatin is accessible [35].
- HNF1A, another transcription factor of importance in B cell development [36].

Of the TFBS that were uniquely identified for the MVA-fHbp boost group, two corresponded to transcription factors with particular relevance to the vaccine-induced response: ETV4 binds the promoter of the Ad E1A gene [37] and CEBPD enhances transcription of IL-6 [38]. The differential usage of TFBS following these heterologous prime-boost vaccine combinations, particularly those with epigenetically-regulated accessibility, implies that the prime vaccine may render particular portions of chromatin accessible in such a manner as to augment or restrict the gene expression changes underlying the immune response to the subsequent boost vaccine. The identification of several of the transcription factors with roles in B cell lymphopoiesis associated with the Ad-fHbp boost suggests a potential role for these pathways in the humoral response induced by this vaccine strategy.

## Discussion

Here we elucidate the distinct molecular pathways that underlie early immune responses to two different viral vector vaccine platforms, administered as a single dose or as part of a heterologous prime-boost combination. Whole blood transcriptomics, performed on samples taken 24 h after the final dose of vaccine, proved sensitive enough to detect changes in gene expression in response to two different transgene products expressed from the same HuAd5 vector. The side-by-side comparison of gene expression data from HuAd5- and MVA-immunised mice revealed differences in both the magnitude and the nature of the transcriptional perturbations induced by these vaccines following intramuscular immunisation. Finally, the comparative approach incorporating the data from mice 24 h after a heterologous boost expanded on these results to demonstrate distinct patterns of gene regulation associated with Ad-fHbp immunisation. These data contribute to the current understanding of the mechanisms of action of viral vector vaccines, underexplored in the field of transcriptomics.

The stimulation of TLR pathways and the downstream MyD88:Mal cascade observed for the HuAd5 constructs tested here is in agreement with what was observed in in vivo studies highlighting the central role of TLR signalling via the MyD88 adaptor in the Ad vector-triggered innate immune response [39, 40]. Additionally, the pathway ORAs performed here reveal extensive enrichment of DNA methylation and acetylation pathways. This may be a host response to the curtailment of viral infection, as epigenetic modification has been studied in the context of DNA virus oncogenesis [41], but vaccine-induced changes to the epigenome of immune cells have gained attention in recent years as part of the emerging concept of ‘innate immune memory’. [42] Further to the characterisation of the vector-induced responses, transgene product-specific pathways were elucidated by contrasting the transgene-encoding Ad constructs with an empty vector. This comparison demonstrated enrichment of protein transport pathways associated with transgene expression as well as antigen-specific TLR stimulation, type I IFN production, enrichment of CD4^+^ T cell signalling, and BCR signalling, all of which function in the initiation of a humoral response [43]. Among the TLRs activated by the delivery of exogenous antigens were TLR9, which plays a role in the activation of pDCs, and TLR2, which drives NF-κB activity, both of which strongly influence the nature of the adaptive immune response through inflammatory signals [44]. Further cellular and immunological analyses are required to support these observations and to validate their functional effects on downstream immune responses elicited by immunisation.

Investigative viral vector vaccine regimens are typically administered as heterologous prime-boost combinations in clinical trials, with the rationale of inducing stronger immune response following exposure to the same antigen while avoiding the possibility of this being dampened by a neutralising and/or cellular memory response to the vector [1]. Priming with an Ad and boosting with an MVA have become the orthodox approach to prime-boost regimens, used in multiple clinical trials to elicit strong cytotoxic responses to intracellular pathogens [15–19]; the heterologous Ad26.ZEBOV prime, MVA-BN-Filo boost vaccine regimen against Ebola has recently received marketing authorisation in Europe [45]. However, unpublished observations in mice and the results of recent studies of heterologous prime-boost combinations of these viral vector-based Ebola vaccines have demonstrated that robust cellular and humoral immunity can be achieved by reversing the order in which they are administered – priming with MVA and boosting with Ad – although the initial humoral immune response to the MVA prime is low, thus limiting the potential utility of this strategy in an outbreak setting [46, 47]. A comparison was made between these two prime-boost strategies in the present study to define the transcriptional basis for the differential immunogenicity between them. While it appears that the limited immune response elicited by MVA is reflected in the gene expression data, with the number of significantly DEGs approximately four-fold lower than Ad at 24 h, the transcriptional changes following an MVA boost are similar to the single dose, while the Ad prime induces almost double the number of significantly DEGs as it does as a single dose. This would suggest that an MVA prime appears to augment the molecular response to a subsequent Ad boost to a greater degree than an Ad prime influences an MVA boost. Further characterisation of transcriptional differences between the prime-boost strategies revealed substantial changes to the host epigenetic profile following the MVA-fHbp/Ad-fHbp regimen, as had been observed for the single dose of Ad-fHbp. These epigenetic changes appear to be reflected in the differential usage of TFBS between the two combinations, but further epigenetic profiling would be required to make conclusions based on these observations and attribute markers to specific cell populations. The MVA/Ad prime-boost also appeared to induce greater IFN-γ signalling, stimulation of preferential TLRs, and antigen processing and presentation compared with Ad/MVA.

The primary limitation of this study is the lack of further cellular analyses to validate the antigen-specific immune responses alluded to in the pathway analyses. This limits the interpretation of these observations and their potential effects on shaping the downstream adaptive immune responses. Characterisation of the early vaccine-induced immune responses that underpin viral vector vaccine prime and boost doses will benefit the development of potent new vaccines against a range of diseases by identifying transcriptional programmes that promote immune cell engagement to enhance immunogenicity and the data presented here should serve as a valuable resource to guide their further exploration in clinical studies.

## Conclusion

Taken together these data demonstrate that the choice of viral vector is important consideration as it determines the nature of the early immune response against the transgene-encoded antigen and that whether a particular vector is given as a prime or a boost further also effects the overall vaccine-induced response. These data will inform the rational design of viral vector vaccines and the order of heterologous prime-boost combinations based on the immune response needed to offer protection against a given disease.

## Materials & methods

### Mouse procedures

All procedures were performed in accordance with the terms and conditions of the UK Home Office Animals Act Project License. Procedures were approved by the University of Oxford Animal Care and Ethical Review Committee. The study was carried out in compliance with the ARRIVE guidelines [48]. Immunisations and cardiac bleeds were performed under general anaesthesia – 3.5% isofluorane mixed with 2 L/min of O^2^. All mice were female C57BL/6 (Harlan) and aged 6–8 weeks at the beginning of experiment. Vaccines were administered by intramuscular injection. Mice received one tenth of the indicated human dose of vaccine – 1 × 10^9^ viral particles for Ad, 1 × 10^7^ viral particles for MVA – as is standard for pre-clinical vaccine evaluation in the mouse immunisation model. The vaccines and corresponding doses are outlined in Table 1 (*n* = 6 per vaccine group). All vaccines were generated by the Viral Vector Core Facility (VVCF, Jenner Institute, University of Oxford). For prime-boost regimens, the booster dose of vaccine was administered 8 weeks (D28) after the prime dose, the standard interval for Ad MVA regimens. Terminal bleeds were performed 24 h after the final dose of vaccine (D1 post-immunisation for single dose groups, D29 post-first dose for prime-boost groups). Blood was collected in pre-filled RNAlater™ (Life Technologies) blood collection microcentrifuge tubes.

**Table 1:** Vaccines and corresponding doses

Vaccine	Dose
Ad-fHbp	D0: 1 × 10^9^ viral particles (50 μL)
Ad-empty	D0: 1 × 10^9^ viral particles (50 μL)
Ad-prefusion F (preF)	D0: 1 × 10^9^ viral particles (50 μL)
MVA-fHbp	D0: 1 × 10^7^ viral particles (50 μL)
MVA-fHbp prime, Ad-fHbp boost	D0: 1 × 10^7^ viral particles (50 μL) MVA-fHbp D28: 1 × 10^9^ viral particles (50 μL) Ad-fHbp
Ad-fHbp prime, MVA-fHbp boost	D0: 1 × 10^9^ viral particles (50 μL) Ad-fHbp, D28: 1 × 10^7^ viral particles (50 μL) MVA-fHbp

### RNA-sequencing

RNA-stabilised blood samples (*n* = 6 per vaccine group, 6 vaccine groups, *n* = 36 in total) were spun at ≥15,000 x *g* for 3 mins in a microcentrifuge and the supernatant was removed and discarded by pipetting. RNA was extracted from mouse whole blood pellets using a Mouse RiboPure™-Blood RNA Isolation Kit (Ambion) according to manufacturer’s instructions. The ribodepleted fraction was selected from the total RNA before conversion to cDNA (Ribo-Zero Plus rRNA Depletion Kit [Illumina]). cDNA was then end-repaired, A-tailed and adapter-ligated prior to amplification (TotalPrep™-96 RNA Amplification Kit [Illumina]), uridine digestion, 75 bp size-selected, and multiplexed. After final QC, samples were paired end sequenced over seven lanes of a flow cell using an Illumina HiSeq4000 instrument.

### Computational methods

Raw FASTQ-formatted reads were assessed for sequence quality using FastQC (https://www.bioinformatics.babraham.ac.uk/projects/fastqc/). Sequences were assessed based on Phred nucleotide quality scores, GC content, number of over-represented sequences (such as globin or ribosomal), and k-mer content. Poor quality sequences were trimmed, where necessary, using Trimmomatic v0.3.3 [49]. HISAT2 v2.1.0 [50] was used for the probabilistic alignment of QC’d FASTQ files to the *Mus musculus* reference genome – GRCh38 release 96 – and the resultant SAM file was sorted by coordinate into a BAM file using the SAMtools (v0.1.18) algorithm [51]. Transcript-based quantification was performed using StringTie v1.3.5 [52] to generate a sample-specific GTF from the GRCh38 *Mus musculus* reference annotation GTF. Sample GTFs were consolidated into a single count matrix by running the Python prepDE.py script (http://ccb.jhu.edu/software/stringtie/dl/prepDE.py).

Count matrix files were read into the latest version of R (4.0.3) using the read.csv function. Counts were filtered using the edgeR [53] filterByExpr function. Linear models were fitted to the data using the lmfit function, and a trended empirical Bayes (eBayes) method was applied to account for errors in the log_2_ fold change (LFC) estimations [54]. Significantly DEGs were defined as those with a false-discovery rate (FDR) adjusted *p*-value, calculated using the Benjamini-Hochberg method, of < 0.01, and an absolute LFC greater than 0.58.

Lists of significantly DEGs for each coefficient were exported using the topTable function, sorting by FDR, for subsequent pathway enrichment analyses. These were conducted using Ensembl gene identifiers with corresponding LFC and FDR values and uploaded to InnateDB [55] to assess enrichment of terms from the Reactome (to assess hallmark immunological responses), INOH (to assess cell signalling pathway activation), and TFBS (to transcription factor enrichment) curated databases. The hypergeometric algorithm and Benjamini-Hochberg method were selected for the ORA and pathway significance correction, respectively. Full tables of InnateDB results for each vaccine group are provided in Supplementary Tables 1, 2, 3, 4, 5, 6, 7, 8, 9, 10, 11 and 12.

## Supplementary Information

**Additional file 1: Supplementary Figure 1.** Overlap between significantly differentially expressed genes (DEGs) in mouse whole blood 24 h after immunisation with human adenovirus serotype 5 (HuAd5) encoding a bacterial (factor H binding protein, Ad-fHbp), viral (pre-fusion F protein, Ad-preF), or no antigen (Ad-empty) and a modified vaccinia Ankara (MVA) virus encoding fHbp. Significantly DEGs were defined as those with an FDR < 0.01. Genes unique to each vaccine group represent putative transgene-encoded antigen-specific genes, while genes in the intersection between all three groups adenovirus groups represent HuAd5 vector-specific genes. Genes overlapping between Ad-fHbp and MVA-fHbp are highlighted as these serve as confirmation of the ability to detect a transgene product-specific gene signature from each vaccine platform. **Supplementary Table 1.** Ad-empty gene ontology over-representation analysis results. **Supplementary Table 2.** Ad-fHbp gene ontology over-representation analysis results. **Supplementary Table 3.** Ad-preF gene ontology over-representation analysis results. **Supplementary Table 4.** MVA-fHbp gene ontology over-representation analysis results. **Supplementary Table 5.** Ad-fHbp prime MVA-fHbp boost gene ontology over-representation analysis results. **Supplementary Table 6.** MVA-fHbp prime Ad-fHbp boost gene ontology over-representation analysis results. **Supplementary Table 7.** Ad-empty pathway over-representation analysis results. **Supplementary Table 8.** Ad-fHbp pathway over-representation analysis results. **Supplementary Table 9.** Ad-preF pathway over-representation analysis results. **Supplementary Table 10.** MVA-fHbp pathway over-representation analysis results. **Supplementary Table 11.** Ad-fHbp prime MVA-fHbp boost pathway over-representation analysis results. **Supplementary Table 12.** MVA-fHbp prime Ad-fHbp boost pathway over-representation analysis results.

## Data Availability

The RNA-seq data have been deposited in the NCBI GEO database under the accession number GSE139529. All correspondence including requests for materials should be addressed to Dylan Sheerin at sheerin.d@wehi.edu.au
